# Clonal expansion of shared T cell receptors reveals the existence of immune commonality among different lesions of synchronous multiple primary lung cancer

**DOI:** 10.1007/s00262-024-03703-8

**Published:** 2024-04-26

**Authors:** Yadong Wang, Zhicheng Huang, Bowen Li, Jianchao Xue, Chao Guo, Zhongxing Bing, Zhibo Zheng, Yang Song, Yuan Xu, Guanghua Huang, Haochen Li, Xiaoqing Yu, Yankai Xia, Ruirui Li, Xiaoyan Si, Li Zhang, Ji Li, Lan Song, Yuanyuan Xiong, Dejian Gu, Mengmeng Song, Zhipeng Zhou, Rongrong Chen, Zhe Feng, Zhixin Bie, Xiaoguang Li, Huaxia Yang, Shanqing Li, Naixin Liang

**Affiliations:** 1 Department of Thoracic Surgery, Peking Union Medical College Hospital, Chinese Academy of Medical Sciences and Peking Union Medical College, Beijing, China; 2 Department of Cardiothoracic Surgery, Civil Aviation General Hospital, Beijing, China; 3 Department of Pulmonary and Critical Care Medicine, Peking Union Medical College Hospital, Chinese Academy of Medical Sciences and Peking Union Medical College, Beijing, China; 4 Department of Pathology, Peking Union Medical College Hospital, Chinese Academy of Medical Sciences and Peking Union Medical College, Beijing, China; 5 Department of Radiology, Peking Union Medical College Hospital, Chinese Academy of Medical Sciences and Peking Union Medical College, Beijing, China; 6 Geneplus-Beijing, Beijing, China; 7 Department of Cardiothoracic Surgery, The Sixth Hospital of Beijing, Beijing, China; 8 Minimally Invasive Tumor Therapies Center, Beijing Hospital, National Center of Gerontology, Institute of Geriatric Medicine, Chinese Academy of Medical Sciences, Beijing, China; 9 State Key Laboratory of Complex Severe and Rare Diseases, Peking Union Medical College Hospital, Chinese Academy of Medical Sciences and Peking Union Medical College, Beijing, China; 10 Department of Rheumatology and Clinical Immunology, National Clinical Research Center for Dermatologic and Immunologic Diseases, Peking Union Medical College Hospital, Chinese Academy of Medical Sciences and Peking Union Medical College, The Ministry of Education Key Laboratory, Beijing, China

**Keywords:** Multiple primary lung cancer, Mutational landscape, T cell receptor, Immune commonality, Therapeutic perspective

## Abstract

The increase in the detection rate of synchronous multiple primary lung cancer (MPLC) has posed remarkable clinical challenges due to the limited understanding of its pathogenesis and molecular features. Here, comprehensive comparisons of genomic and immunologic features between MPLC and solitary lung cancer nodule (SN), as well as different lesions of the same patient, were performed. Compared with SN, MPLC displayed a lower rate of EGFR mutation but higher rates of BRAF, MAP2K1, and MTOR mutation, which function exactly in the upstream and downstream of the same signaling pathway. Considerable heterogeneity in T cell receptor (TCR) repertoire exists among not only different patients but also among different lesions of the same patient. Invasive lesions of MPLC exhibited significantly higher TCR diversity and lower TCR expansion than those of SN. Intriguingly, different lesions of the same patient always shared a certain proportion of TCR clonotypes. Significant clonal expansion could be observed in shared TCR clonotypes, particularly in those existing in all lesions of the same patient. In conclusion, this study provided evidences of the distinctive mutational landscape, activation of oncogenic signaling pathways, and TCR repertoire in MPLC as compared with SN. The significant clonal expansion of shared TCR clonotypes demonstrated the existence of immune commonality among different lesions of the same patient and shed new light on the individually tailored precision therapy for MPLC.

**Supplementary Information:**

The online version contains supplementary material available at 10.1007/s00262-024-03703-8.

## Introduction

Lung cancer remains the leading cause of cancer-associated deaths worldwide [1]. With the widespread application of computed tomography in lung cancer screening, the detection rate of synchronous multiple primary lung cancer (MPLC) is continually increasing in clinical practice [2]. The underlying mechanisms associated with the initiation and development of MPLC remain unknown. Compared to solitary lung cancer nodule (SN), MPLC has posed critical challenges to clinicians in the fields of diagnosis and treatment [3].

The introduction of next-generation sequencing (NGS) has greatly broadened our understanding of MPLC, particularly in the differential diagnosis between MPLC and intrapulmonary metastasis [4]. Genomic analyses revealed substantial heterogeneity in the mutational landscape between different lesions of the same patient with MPLC, while metastatic lesions always harbored the same driver mutations compared to the primary tumor [5]. Many investigators consider that the occurrence of MPLC is a consequence of multiple independent and parallel evolutionary events [6]. However, given the shared germline mutations and environmental exposure, there might be an unexplored link among different lesions of the same patient with MPLC.

The latest research reveals that air pollution promotes lung cancer by acting on healthy lung cells with pre-existing oncogenic mutations, indicating genomic alterations and environmental exposure act synergistically in promoting tumorigenesis [7]. In addition, the proliferation and metastasis of tumor are shaped by the interaction between tumor cells and host immune surveillance. T cells can recognize tumor-associated antigens via the T cell receptor (TCR) and in turn play an important role in antitumor immunity [8]. Several studies have revealed that TCR sequencing could provide valuable information about the diagnosis, prognosis, and prediction of treatment response in lung cancer [9, 10]. Therefore, systematically assessing genomic alterations and TCR repertoire of MPLC and comparing them with those of SN will further deepen our understanding of MPLC.

In the current study, targeted NGS using a 1,021-gene panel was performed to assess the mutational landscape and tumor mutation burden (TMB). In parallel, the activation status of antitumor immune response was evaluated using programmed cell death-ligand 1 (PD-L1) expression and TCR repertoire. Comprehensive comparisons across genomic and immune features between MPLC and SN were performed. Furthermore, the relationship between different lesions of the same patient with MPLC was explored.

We aim for this work to broaden our knowledge of MPLC, particularly focusing on its immunological features, and attract more researchers to actively delve into the research of this increasingly prevalent type of lung cancer.

## Materials and methods

### Patient cohorts

A total of 151 patients with lung cancer who underwent surgery from February 2017 to October 2020 were enrolled in this retrospective study. Of these, 27 patients with a total of 69 lesions were diagnosed as synchronous MPLC based on the American College of Chest Physicians (ACCP) guidelines [11] and following thorough deliberation by a multidisciplinary team consisting of thoracic surgeons (YW, ZH, BL, SL, NL), respiratory oncologists (XS, LZ), a radiologist (LS), a pathologist (JL), and molecular biologists (YX, RC). When the imaging or pathology diagnosis was inconclusive, MPLC was defined if the following criteria were met: (1) no more than two mutations were shared between different lesions [12]; (2) no recurrence or death was observed at the 36-month follow-up examination. The remaining 124 patients were diagnosed with SN. Tumor staging was evaluated based on the 8th edition of the American Joint Committee on Cancer TNM staging system for lung cancer. Patients with a documented history of autoimmune disorders, prior receipt of antitumor therapy before surgery, or postoperative suspicion of intrapulmonary metastasis were excluded. This study was conducted in accordance with the Declaration of Helsinki. Ethical approval was obtained from the ethical committee of Peking Union Medical College Hospital (S-K1670), and all patients provided written informed consent at the time of enrollment.

### DNA extraction, targeted capture and next-generation sequencing

Specimen collection, processing, and mutational analysis were conducted as previously described [13]. Briefly, the DNA from formalin-fixed, paraffin-embedded specimens was isolated using Maxwell®16 FFPE Plus LEV DNA Purification kit (Qiagen). The libraries were sequenced on a NextSeq CN 500 system (Illumina, San Diego, CA) after hybridization to custom-designed biotinylated oligonucleotide probes (Roche NimbleGen, Madison, WI) targeting 1,021 tumor-related genes [14].

After the removal of terminal adaptor sequences and low-quality reads with FASTP, the remaining reads were mapped to the reference human genome (hg19) and aligned using the Burrows-Wheel Aligner (0.7.12-r1039) with default parameters. Duplicated reads were removed with the MarkDuplicates tool in Picard (4.0.4.0; Broad Institute, Cambridge, MA, USA). Realdcaller (1.8.1; Geneplus-Beijing, inhouse) and GATK (v3.6–0-g89b7209; Broad Institute) were employed to detect tumor somatic single nucleotide variants and small insertions and deletions. Contra (2.0.8) was used to identify copy number variations. NCsv (0.2.3; Geneplus-Beijing, inhouse) was employed to detect structural variants. All candidate variants were manually confirmed by using the integrative genomics viewer browser. Variants were filtered to exclude germline mutations in dbSNP and those that occur at a population frequency of > 1% in the Exome Sequencing Project. An inhouse database of clonal hematopoiesis variants of > 10,000 pan-cancer patients and healthy individuals was used to filter clonal hematopoiesis-related variants.

### Evaluation of TMB and PD-L1 expression

TMB was calculated as the total number of somatic non-synonymous mutations per mega-base of the genome examined. Tumor PD-L1 expression was determined by the percentage of cells expressing PD-L1 at any intensity above background staining using the PD-L1 IHC 22C3 pharmDx assay (Agilent Technologies, Santa Clara, CA, USA).

### TCR sequencing and assessment of the TCR repertoire

All lesions from the two cohorts included in this study passed quality control, and TCR sequencing was performed as previously described, with details provided below [15]. Multiplex PCR amplification of the CDR3 region of the TCR β chain was conducted including PCR1 and PCR2, inclusively and semi-quantitatively. Multiplex PCR1 assay was conducted by using a set of 32 V forward and 13 J reverse primers to amplify V(D)J combinations. Universal primers were used for the second round of multiplex PCR. Sequencing libraries were loaded onto an Illumina HiSeq X ten system, and 151-bp-length reads were obtained. The CDR3 sequence was defined as the amino acids between the second cysteine of the V region and the conserved phenylalanine of the J region, according to the ImMunoGeneTics V, D, and J gene references. The CDR3 sequences were identified and assigned using the MiXCR software package. TCR repertoire parameters in this study were calculated after normalizing TCR read counts to 1,000,000 for all samples to ensure comparability between different lesions.

As previously reported, the number of distinct TCR clonotypes is calculated as the total number of T cells divided by the mean number of cells per clonotype [16]. Shannon index (Shannon’s entropy) is defined as:

$$\mathrm{Shannon index}= -\sum_{i=1}^{N}{\text{pilnpi}}$$

where *pi* represents the ratio of sequence *i* to the total *N* sequences [8]. More diverse distribution of CDR3 sequences is reflected by a higher Shannon index. Clonality can be calculated by 1—(Shannon index/ln *k*), where *k* is the number of productive unique sequences. Values of clonality close to 0 represent an extremely even distribution of different clones (polyclonal), whereas values approaching 1 correspond to a distinct asymmetrical distribution (monoclonal). The frequency of top 100 clones is calculated as the sum of the frequencies of the top 100 ranked TCR clones in each lesion. The similarity and overlap of TCR repertoire between every two lesions were quantified using the Morisita overlap index (MOI), which takes into account not only the specific rearrangements but also their respective frequencies. The MOI ranges from 0 to 1, with 1 indicating identical TCR repertoires and 0 indicating completely distinct ones. Shared TCR clonotypes refer to TCR clonotypes that are present in two or multiple lesions of the same patient with MPLC, and the proportion of shared TCR clonotypes in each lesion is calculated as the number of shared TCR clonotypes divided by the total number of TCR clonotypes present in the corresponding lesion.

### Statistical analysis

The analyses described here were all pre-planned. Descriptive statistics were used to compare demographic and clinical characteristics between cohorts. We subsequently plan to conduct comprehensive comparisons across mutational and immune features, including gene mutation status, TMB, PD-L1 expression, and TCR repertoire, between the MPLC and SN cohorts. Prespecified subgroup analyses of TCR repertoire in lesions of invasive adenocarcinoma between the two cohorts were planned due to the higher risk of metastasis in invasive lung adenocarcinoma. We also plan to investigate the relationship between TCR repertoire and the four pathological subtypes of lung adenocarcinoma, as well as TMB. Following these, our focus will be on exploring the homogeneity and heterogeneity among two or multiple lesions of the same patient in the MPLC cohort, including the proportion of shared TCR clonotype and corresponding clonal expansion status in each lesion.

Continuous variables were described using median and interquartile range (IQR), and categorical variables were reported with numbers and percentages. For comparisons between two groups, the Mann-Whitney U test or Student’s t test was used as appropriate. For comparisons between multiple groups, one-way analysis of variance (ANOVA) and the Kruskal–Wallis test was employed for normally and non-normally distributed variables, respectively. Categorical variables were analyzed using the Chi-squared test or Fisher’s exact test, where appropriate. Correlations between continuous variables were assessed using Spearman correlation coefficient. All statistical tests were two-sided, and *p*-values of less than 0.05 were considered significant. Statistical analysis was performed using R software, version 4.0.2.

## Results

### Clinical and pathological characteristics

**Fig. 1:**
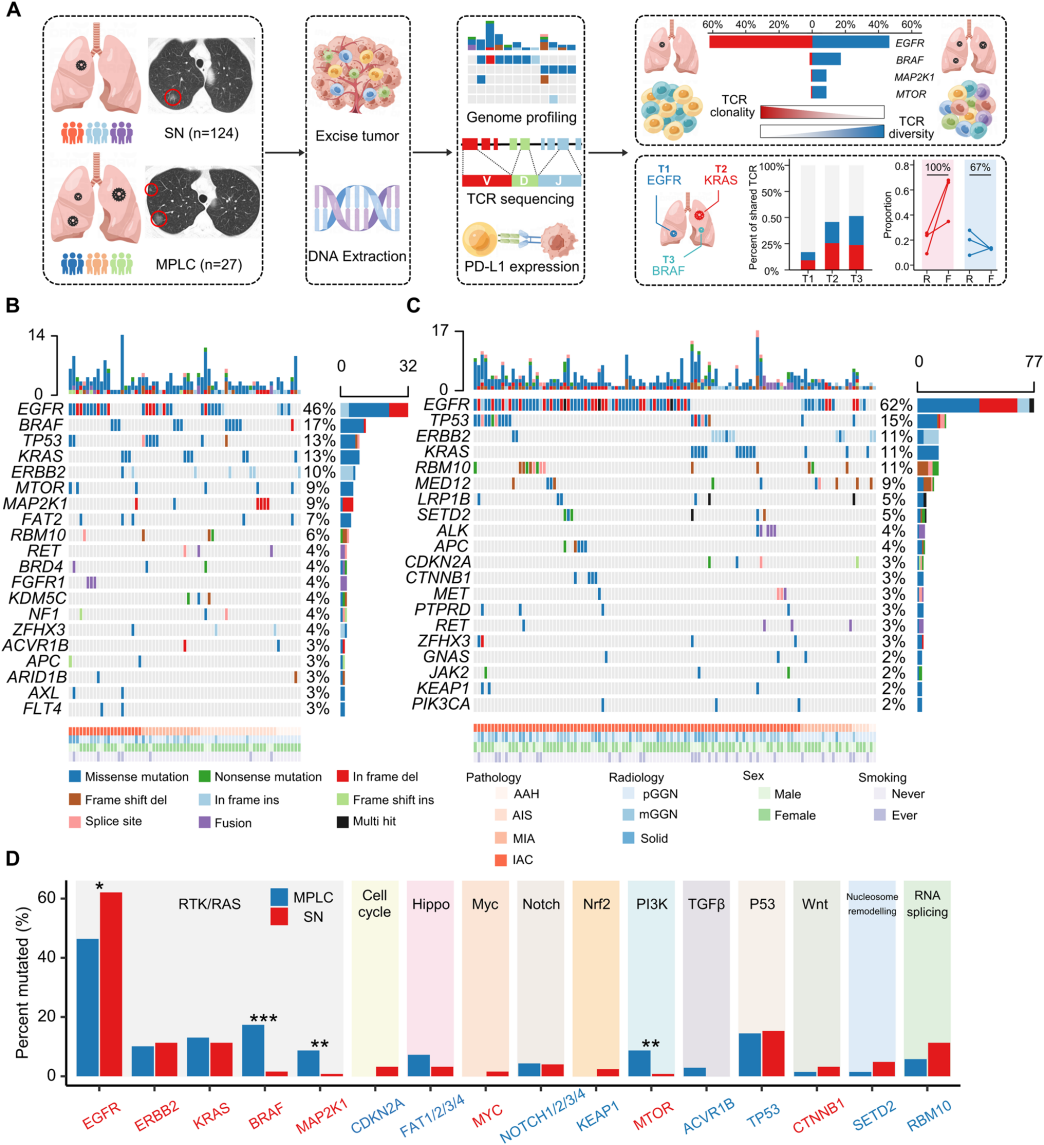
Mutational landscape and oncogenic signaling pathways in MPLC and SN. The overall design of this study (**A**). Mutational landscapes of top 20 non-synonymous somatic mutations in MPLC (**B**) and SN (**C**). Each column represents one lesion, and each row represents one gene. Samples displayed as columns are ordered by the histological subtypes, and gene mutations are shown in different colors according to the type of alterations. The number of somatic mutations and mutation rate of each gene are shown at the top and right of the figure, respectively. Comparison of the mutation rate of key genes in lung cancer-associated oncogenic signaling pathways between MPLC and SN (**D**). Oncogenes labeled in red and tumor suppressor genes labeled in blue are listed on the horizontal axis ordered by the signaling pathways. The vertical axis represents the mutation rates obtained from different cohorts. *MPLC*, Multiple primary lung cancer; *SN*, Solitary lung cancer nodule; *AAH*, Atypical adenomatoid hyperplasia; *AIS*, Adenocarcinoma in situ; *MIA*, Minimally invasive adenocarcinoma; *IAC*, Invasive adenocarcinoma; *pGGN*, Pure ground-glass nodule; *mGGN*, Mixed ground-glass nodule. *P < 0.05, **P < 0.01, ***P < 0.001 (Chi-squared/Fisher’s exact test)

In this study, 27 patients with synchronous MPLC and 124 patients with SN were included (Fig. 1A), and the detailed clinicopathological information is presented in Table 1. The majority (*n* = 19, 70%) of patients with MPLC had less than three lesions, and the specific reasons for the diagnosis of MPLC in each patient were illustrated in Supplementary Table 1. In the MPLC cohort, the median age was 57 (IQR, 51–64.5) years, with 77.8% (*n* = 21) females and 88.9% (*n* = 24) never smokers. According to the TNM staging, 70.4% (*n* = 19) of patients were stage I. Correspondingly, the median age was 58.5 (IQR, 49–64) years in the SN cohort, with 67.7% (*n* = 84) females, 78.2% (*n* = 97) never smokers, and 87.1% (*n* = 108) stage I diseases.

**Table 1:** Clinical and pathological characteristics of 27 patients with synchronous MPLC and 124 patients with SN

Characteristics	MPLC (*n* = 27)	SN (*n* = 124)	*P* value
Age, years			0.51^a^
Median (IQR)	57 (51–64.5)	58.5 (49–64)	
Gender, *n* (%)			0.36^b^
Male	6 (22.2)	40 (32.3)	
Female	21 (77.8)	84 (67.7)	
Smoking, *n* (%)			0.29^b^
Never	24 (88.9)	97 (78.2)	
Ever	3 (11.1)	27 (21.8)	
Tumor stage, *n* (%)			0.07^b^
AAH	1 (3.7)	2 (1.6)	
AIS	5 (18.5)	6 (4.9)	
I	19 (70.4)	108 (87.1)	
II	1 (3.7)	4 (3.2)	
III	1 (3.7)	4 (3.2)	
Histological type, *n* (%)	(*n* = 69)	(*n* = 124)	< 0.001^b^
AAH	9 (13.1)	2 (1.6)	
AIS	22 (31.9)	6 (4.8)	
MIA	17 (24.6)	18 (14.5)	
IAC	21 (30.4)	98 (79.1)	
Radiology, *n* (%)			0.06^b^
pGGN	50 (72.5)	68 (54.8)	
mGGN	13 (18.8)	38 (30.7)	
Solid	6 (8.7)	18 (14.5)	

*MPLC* Multiple primary lung cancer, *SN* Solitary lung cancer nodule, *IQR* Interquartile range, *AAH* Atypical adenomatoid hyperplasia, *AIS* Adenocarcinoma in situ, *MIA* Minimally invasive adenocarcinoma, *IAC* Invasive adenocarcinoma, *pGGN* Pure ground-glass nodule, *mGGN* Mixed ground-glass nodule, ^a^Mann–Whitney U test, ^b^Chi-squared/Fisher’s exact test

A total of 69 lung cancer or precursor lung cancer lesions were identified in the MPLC cohort, including 50 cases of pure ground-glass nodules (pGGN), 13 cases of mixed ground-glass nodules (mGGN), and 6 cases of solid nodules. According to their histological subtypes, 13.1% (*n* = 9) were atypical adenomatoid hyperplasia (AAH), 31.9% (*n* = 22) were adenocarcinoma in situ (AIS), 24.6% (*n* = 17) were minimally invasive adenocarcinoma (MIA), and 30.4% (*n* = 21) were invasive adenocarcinoma (IAC). Correspondingly, 68 cases were classified as pGGN, 38 cases were classified as mGGN, and 18 cases were classified as solid nodules in the SN cohort. As for the histological subtypes, 1.6% (*n* = 2) were AAH, 4.8% (*n* = 6) were AIS, 14.5% (*n* = 18) were MIA, and 79.1% (*n* = 98) were IAC. On the whole, besides a higher proportion of precursor lung cancer lesions in the MPLC cohort, there were no significant differences observed in the distribution of other baseline features between the two cohorts.

### Differences in the mutational landscape and activation status of oncogenic signaling pathways between MPLC and SN

The mutational landscapes of MPLC and SN are shown in Fig. 1B and C, respectively. The vast majority of lesions were identified to harbor at least one somatic mutation, both in the MPLC (98.6%, *n* = 68) and in the SN (97.6%, *n* = 121) cohort. The histopathological characteristics and mutational profiles of the different lesions within the same patient in the MPLC cohort were included in Supplementary Fig. 1. The most frequently altered gene in the MPLC cohort was *EGFR* (46.4%), followed by BRAF (17.4%), TP53 (13.0%), KRAS (13.0%), ERBB2 (10.1%), MTOR (8.7%), and MAP2K1 (8.7%). Correspondingly, *EGFR* mutation was also frequently identified (62.1%), followed by TP53 (15.3%), ERBB2 (11.3%), KRAS (11.3%), and RMB10 (11.3%) in the SN cohort.

We subsequently analyzed the differences in the mutation rates of key genes in lung cancer-related oncogenic signaling pathways between lesions of MPLC and those of SN [17]. Compared with SN, lesions of MPLC displayed a significantly lower rate of *EGFR* mutation (*p* < 0.05), but significantly higher rates of BRAF (*p* < 0.001), MAP2K1 (*p* < 0.01), and *MTOR* mutation (*p* < 0.01) (Fig. 1D). Intriguingly, genes with different alteration rates between the two cohorts were located in several important cancer-related signaling pathways. Among these, the mitogen-activated protein kinase (MAPK) and phosphoinositide-3-kinase (PI3K) signaling pathways were significantly activated in MPLC, which were the two major signaling pathways that lie downstream of receptor tyrosine kinase (RTK)/Ras (Supplementary Fig. 2). Overall, the mutational landscape and activation status of oncogenic signaling pathways in MPLC were different from those of SN.

### Similar and relatively low mutational load and PD-L1 expression in MPLC and SN

**Fig. 2:**
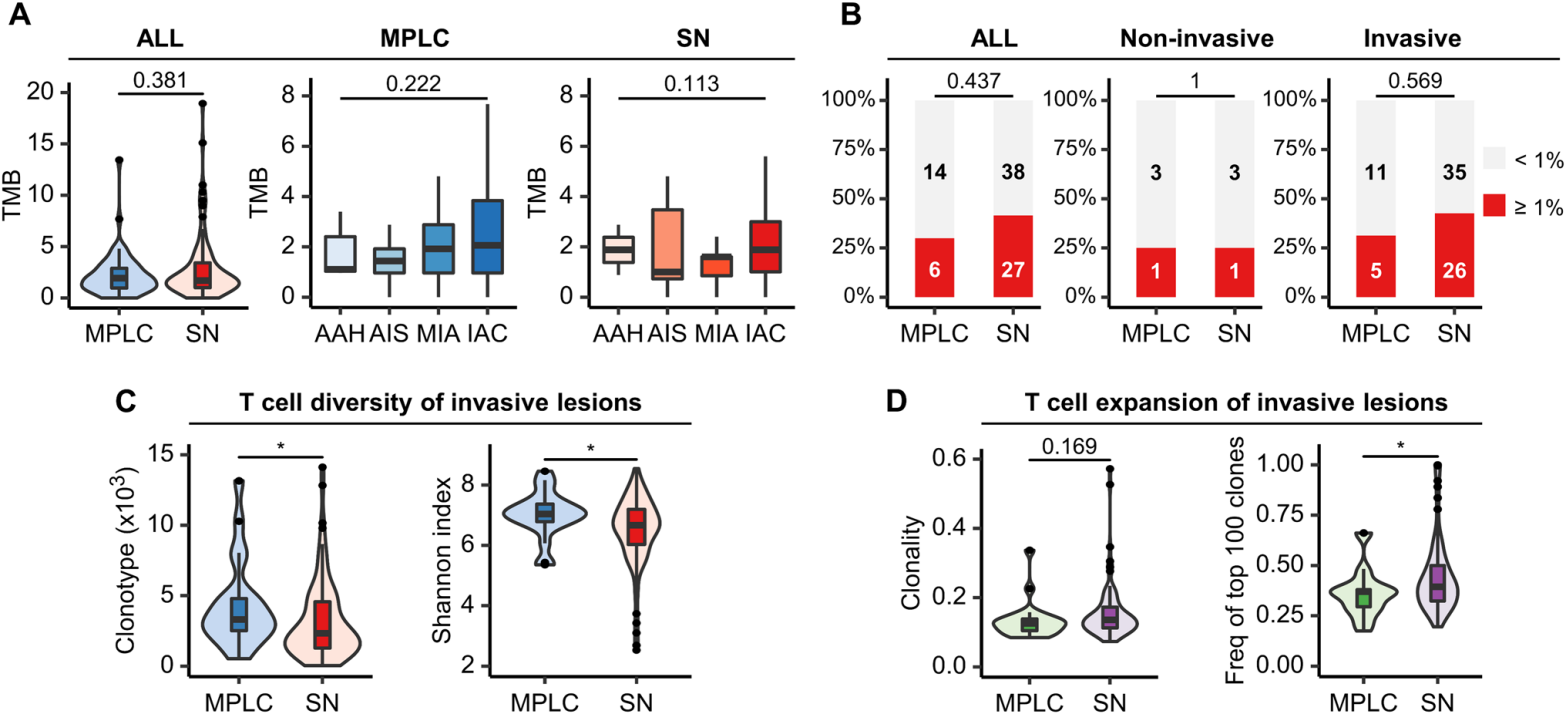
The distribution and differences in TMB, PD-L1 expression, and TCR repertoire between MPLC and SN. Comparison of TMB between lesions of MPLC and SN, and the distribution of TMB across different histological subtypes in the corresponding cohort (**A**). Comparisons of PD-L1 expression in the total, noninvasive, and invasive lesions between MPLC and SN (**B**). Distinct TCR diversity (**C**) and expansion (**D**) in invasive lesions of MPLC and SN. *MPLC*, Multiple primary lung cancer; *SN*, Solitary lung cancer nodule; *TCR*, T cell receptor; *TMB*, Tumor mutation burden; *PD-L1*, Programmed cell death-ligand 1; AAH, Atypical adenomatoid hyperplasia; *AIS*, Adenocarcinoma in situ; *MIA*, Minimally invasive adenocarcinoma; *IAC*, Invasive adenocarcinoma. *P < 0.05, **P < 0.01, ***P < 0.001 (Mann–Whitney U test or Kruskal–Wallis test)

The median TMB was 1.9 mut/Mb (IQR, 1.0–2.9 mut/Mb) in the MPLC cohort and 1.7 mut/Mb (IQR, 1.0–3.4 mut/Mb) in the SN cohort. Overall, TMB was relatively low and no significant difference in TMB was observed between MPLC and SN (Fig. 2A). In light of the evolutionary trajectory of lung adenocarcinoma from preneoplasia AAH, to preinvasive AIS, to microinvasive MIA, and eventually IAC, the relationship between TMB and histopathologic subtypes was further explored. It was shown that no statistically significant differences were noted in TMB among these four subtypes in both MPLC and SN cohorts (Fig. 2A).

PD-L1 expression was assessed in 20 lesions of 10 patients in the MPLC cohort and 65 patients in the SN cohort. The positive rate was 30.0% and 41.5% when 1% was used as a cutoff, respectively. No significant difference was seen in the positive rate of PD-L1 expression between the two cohorts (Fig. 2B). We classified AAH and AIS as noninvasive lesions, while MIA and IAC as invasive lesions to carry out further subgroup analysis. Similarly, differences in PD-L1 expression between the two cohorts were not found in either noninvasive or invasive subgroups (Fig. 2B).

### Distinct TCR repertoire in invasive lesions of MPLC and SN

Given the significant role of T cells in enhancing antitumor immune response, we next investigated the T cell repertoire by performing TCR sequencing and focused on two groups of TCR repertoire metrics, including diversity (clonotype and Shannon index) and expansion (clonality and the frequency of top 100 clones). The median TCR read counts of lesions in the MPLC cohort and SN cohort were 13,297,636 (IQR, 8,507,376–21,848,619) and 10,768,030 (IQR, 9,229,574–16,245,915), respectively, with no significant difference observed between the two cohorts (*p* = *0.299*) (Supplementary Tables 2 and 3). The medians of Shannon index and clonality in lesions of MPLC were 6.7 (IQR, 5.7–7.2) and 0.1 (IQR, 0.1–0.2), respectively (Supplementary Fig. 3A, B). The medians of these two metrics in SN were 6.6 (IQR, 5.8–7.2) and 0.1 (IQR, 0.1–0.2), respectively (Supplementary Fig. 3C, D). The intrapatient differences in TCR repertoire among lesions of the same patient in the MPLC cohort were further quantified as the difference between the maximum and minimum values of TCR metrics divided by the maximum value in the patient. The analysis of TCR Shannon index revealed considerable heterogeneity in the relative differences across different lesions of the same patient in the MPLC cohort, ranging from as large as 48.8% in P7 to as small as 0.8% in P19 (Supplementary Fig. 3E). Similarly, substantial intrapatient differences in TCR clonality were noted (Supplementary Fig. 3F), with variation spanning from 54.3% in P15 to 4.4% in P9.

Further comparisons of TCR repertoire metrics between MPLC and SN showed that no significant differences were observed when all lesions were included in the analyses (Supplementary Fig. 4A, B). However, lesions of invasive adenocarcinoma in the MPLC cohort exhibited significantly higher TCR diversity and lower frequency of top 100 clones than those in the SN cohort (*p* < 0.05). In addition, a tendency toward decreased clonality was observed in the invasive adenocarcinoma subgroup of MPLC, although the difference was not significant (*p* = *0.169*) (Fig. 2C, D). Of note, significant differences in TCR repertoire metrics between MPLC and SN disappeared when lesions of AAH, AIS, and MIA were included in the analyses (Supplementary Fig. 4C, D).

**Fig. 3:**
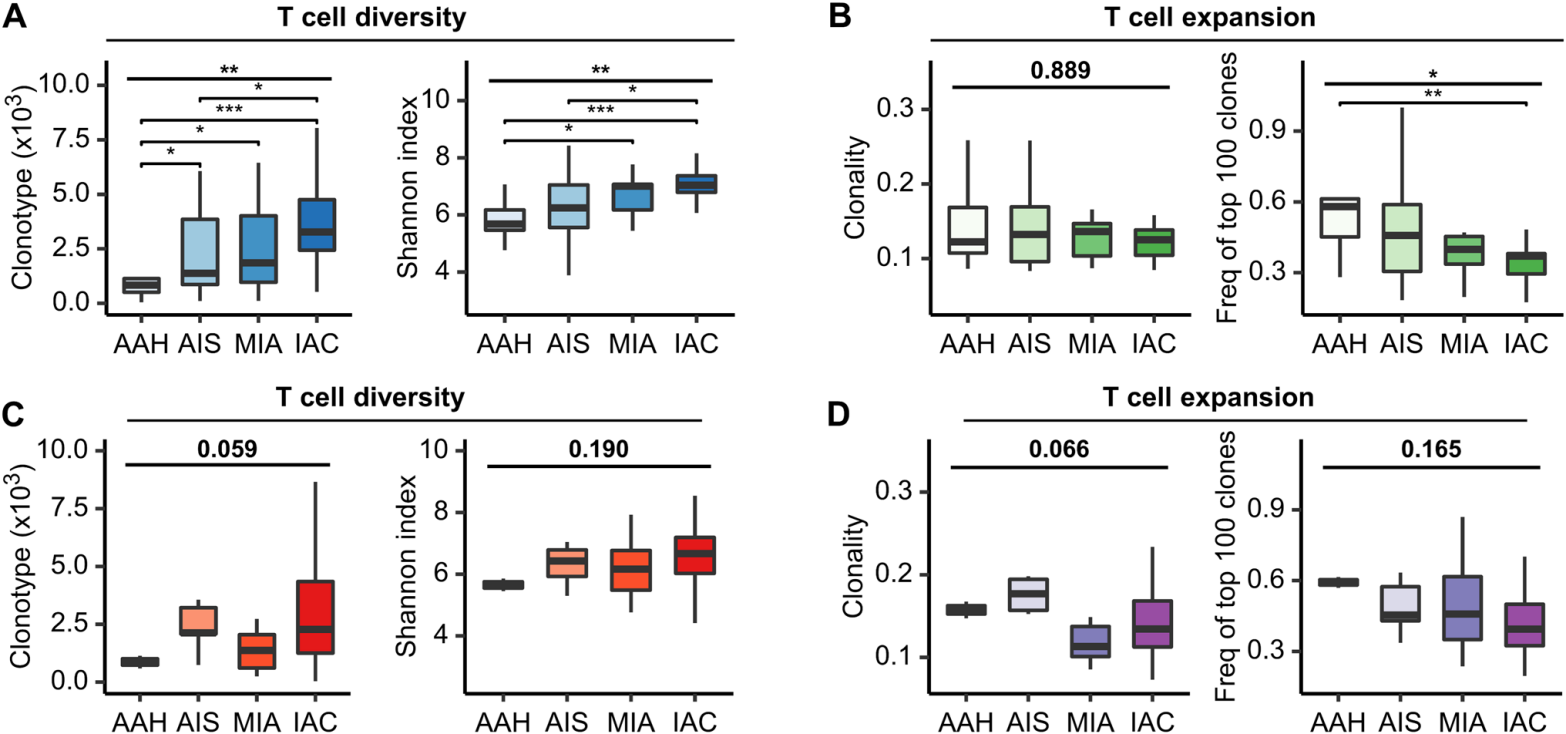
The distribution of TCR repertoire across different histological subtypes in MPLC and SN. The distribution and difference in TCR diversity (**A**) and expansion (**B**) across different histological subtypes in the MPLC cohort. The distribution and difference in TCR diversity (**C**) and expansion (**D**) across different histological subtypes in the SN cohort. *MPLC*, multiple primary lung cancer; *SN*, solitary lung cancer nodule; *TCR*, T cell receptor; *AAH*, atypical adenomatoid hyperplasia; *AIS*, adenocarcinoma in situ; *MIA*, minimally invasive adenocarcinoma; *IAC*, Invasive adenocarcinoma. *P < 0.05, **P < 0.01, ***P < 0.001 (Kruskal–Wallis test)

The invasive growth of tumor is often viewed as a dynamic and plastic process between tumor cells and the microenvironment, leading to a stable balance between the shaping and control of the tumor by the adaptive immune system. TCR sequencing in the MPLC cohort showed that AAH exhibited the lowest diversity (*p* < 0.01), and IAC had the lowest frequency of top 100 clones (*p* < 0.05) (Fig. 3A, B). However, significant differences in trends of TCR repertoire metrics were only observed in MPLC but not in SN (Fig. 3C, D).

### Associations between TMB and TCR repertoire

A high mutation load would lead to higher neoantigen expression and immunogenicity, eventually resulting in increased opportunities for the tumor to be recognized by T cells. It has been shown that high TMB in lung adenocarcinoma is associated with increased clonality of infiltrated T cells and favorable efficacy of immunotherapy [18]. Unexpectedly, significant correlations between TMB and TCR repertoire metrics were not found in the MPLC (Supplementary Fig. 5A, B) nor the SN cohort (Supplementary Fig. 5C, D). One possible explanation was that most lesions were at the early stages with a relatively low TMB and had just begun to evade immune surveillance.

### The heterogeneity and homogeneity of TCR repertoire among multiple lesions of the same patient with MPLC

**Fig. 4:**
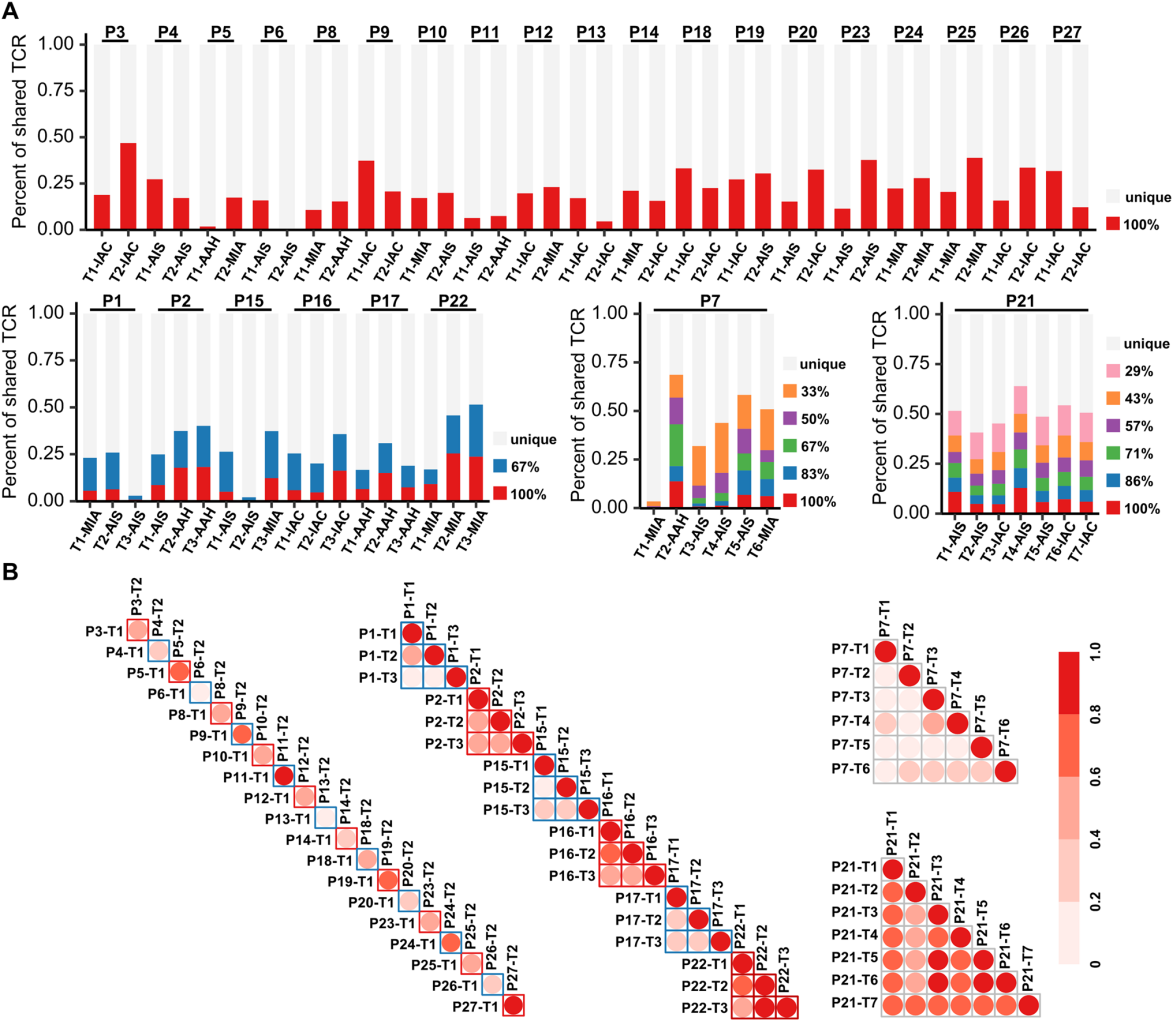
Distribution and similarity of TCR repertoire among different lesions of the same patient in the MPLC cohort. Distribution of shared TCR clones among different lesions of the same patient (**A**). The different colors indicate different degrees of shared TCR clones in each patient. Shared TCR clones between different lesions of the same patient as quantified by the MOI (**B**). Darker colors indicate higher MOI. *MPLC*, multiple primary lung cancer; *TCR*, T cell receptor; *MOI*, Morisita overlap index

Analysis of immune cell infiltration has highlighted the existence of heterogeneity in immune characteristics of different lesions of MPLC [19]. However, detailed studies about TCR repertoire in MPLC are currently lacking, particularly the mutual comparisons among multiple lesions of each patient. To this end, we first investigated the percent of shared TCR in each lesion, which was calculated as the number of shared TCR across lesions of the same patient, divided by the number of self-owned TCR in the corresponding lesion. As shown in Fig. 4A, the vast majority of TCR were found exclusively in single lesion, and the percent of unique TCR in each lesion displayed substantial intrapatient and interpatient heterogeneity. In patients with two primary lung cancer lesions, the highest percentage of shared TCR was 46.9% in P3-T2, and the lowest was 0.5% in P6-T2. In patients with more than two primary lung cancer lesions, P7-T2 harbored the highest percentage of shared TCR of 68.6%, and P15-T2 had the lowest value of 2.1%. Similarly, significant intrapatient and interpatient heterogeneity in MOI was found (Fig. 4B). The maximum and minimum MOI was 0.934 occurring in P11 and 0.008 in P6, respectively.

**Fig. 5:**
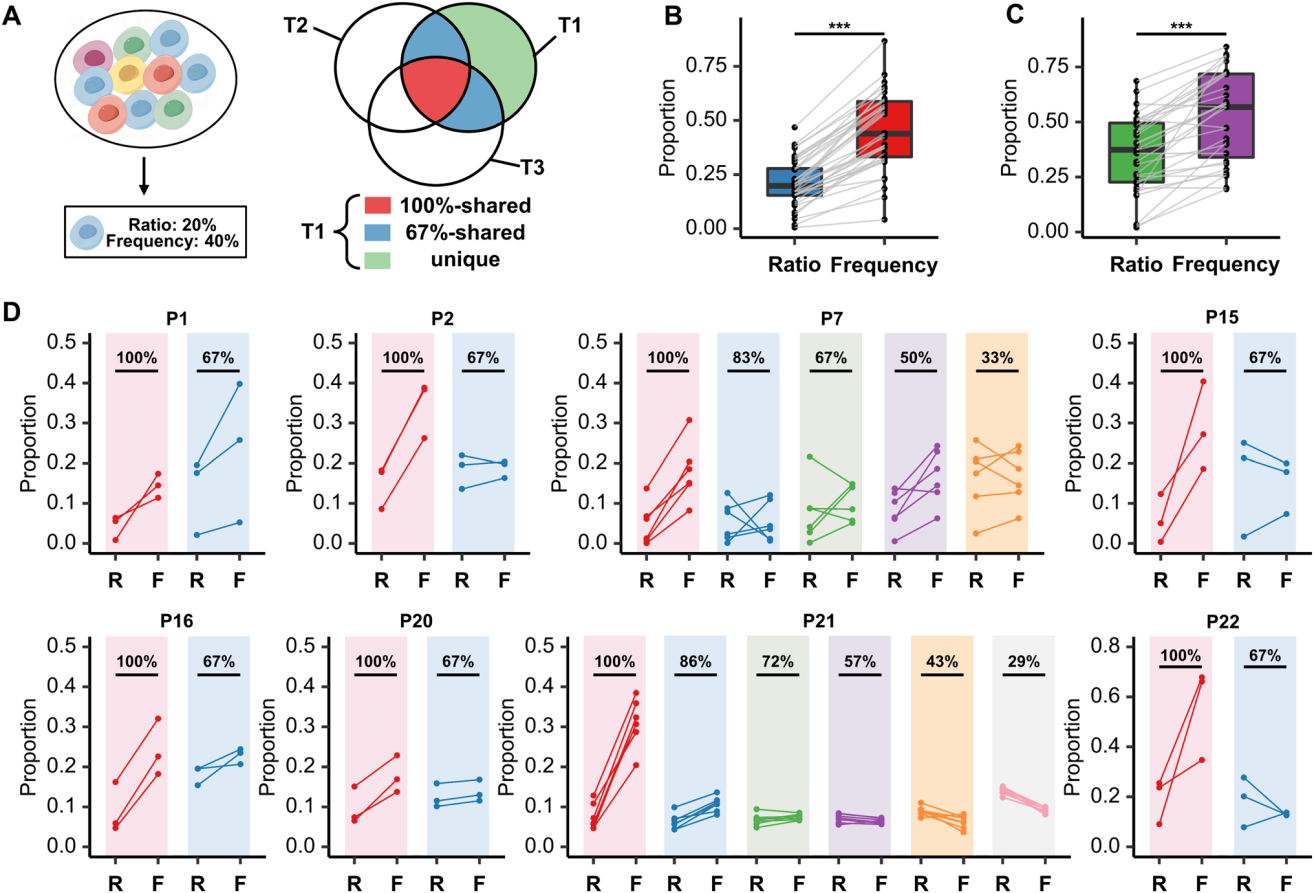
The significant clonal expansion of shared TCR clones among lesions of the same patient in the MPLC cohort. Schematic diagram of the shared TCR (**A**). The significant differences between the ratio and frequency of shared TCR clones in patients with double primary lung cancer lesions (**B**) and in patients with more than two lesions (**C**). The specific distributions of the ratio and frequency of shared TCR clones in patients with more than two lesions (**D**). The different colors indicate different degrees of shared TCR clones in each patient. *MPLC*, multiple primary lung cancer; *TCR*, T cell receptor. *P < 0.05, **P < 0.01, ***P < 0.001 (paired t test)

Considering the identical germline background and environmental exposure shared by lesions of the same patient, it is reasonable to speculate that shared TCR clonotypes might reflect the immune commonality and a certain level of homogeneity among lesions in patient with MPLC. We then investigated the role of shared TCR clonotypes by introducing two new metrics termed the ratio and the frequency of shared TCR clonotypes (Fig. 5A). The former was calculated in a manner similar to the percent of shared TCR clonotypes in each lesion as previously described. Taking P1 with three lesions as an example, TCR clonotypes in P1-T1 were divided into three categories, including 100% shared clonotypes that were found in all three lesions (in red), 67% shared clonotypes that were found in only one of the other two lesions (in blue), and unique clonotypes that were self-owned (in green). The frequency of shared TCR clonotypes was defined as the proportion of the reads of shared TCR clonotypes to the sum reads of all TCR clonotypes in each lesion.

Intriguingly, the frequency of shared TCR clonotypes was significantly higher than the ratio of corresponding TCR clonotypes both in patients with double primary lung cancer lesions (*p* < 0.001) (Fig. 5B) and in patients with more than two lesions (*p* < 0.001) (Fig. 5C). This implied that the shared TCR clonotype harbored clonal expansion advantage when compared with unique TCR clones in each lesion. It is worth noting that the opposite trend between frequency and ratio was seen in a minority of the shared TCR clonotypes in Fig. 5C. Therefore, we further enumerated the distribution of all kinds of shared TCR clonotypes in patients with more than two lesions to explore the intrinsic regularities (Fig. 5D). Of note, the phenomenon that the frequency of shared TCR clonotypes was steadily higher than the ratio of corresponding TCR clonotypes only existed at 100% shared TCR clonotypes.

## Discussion

In recent years, the incidence of MPLC is significantly increased due to the widespread implementation of lung cancer screening [2]. Despite the substantial advances in surgery and radiotherapy, removing all of the lesions may not be feasible for all patients, especially for those with limited pulmonary function or a large number of lung cancer lesions [20, 21]. Consequently, the management of MPLC is much more complex than that of SN and remains an unmet medical need. The pathogenesis of MPLC is currently not well understood. Potentially susceptible germline mutations may serve as early events contributing to the development of a small subset of MPLC, yet further in vivo experimental validation is needed to verify the hypothesis [12]. More extensive research, particularly comparative analyses with SN, is indispensable for advancing our understanding of MPLC. In this study, mutational landscapes and immunologic characterizations of MPLC and SN were comprehensively assessed and compared using various integrative approaches.

The histopathologic and mutational heterogeneity among multiple lesions of the same patient with MPLC observed in our research confirmed their nonmetastatic nature. When it comes to the mutational landscape, one recent study with a large sample size suggested that *EGFR* mutation was more commonly mutated in SN cases, while *BRAF* mutation was more frequently observed in MPLC cases [14]. Similar differences in the mutation rate between MPLC and SN were found in our study, indicating that distinct mutational patterns did exist in MPLC. Notably, differentially mutated genes were exactly located in the upstream and downstream of RTK/RAS signaling pathway [17]. Therefore, MPLC might not be a simple combination of multiple solitary lung cancer lesions due to the influence of identical germline and environmental background. The specific causes and clinical significance of this finding have not been clearly elucidated. Recent studies have indicated that the genetic heterogeneity of MPLC was compatible with the convergent evolution, and only a limiting number of signaling pathways were mutated [22, 23]. However, evolutionary tree reconstruction in another study reached the opposite conclusion indicating that divergent rather than convergent evolution at the levels of both individual genes and functional pathways [24]. Overall, most of the previous research only included gene mutation information and relatively small sample sizes, making it premature to draw any definitive conclusion.

In clinical practice, the approval of adjuvant targeted therapy after surgery in localized lung cancer affords us an opportunity to evaluate the efficacy of targeted therapy in residual lesions in patients with MPLC [25]. However, postoperative *EGFR* tyrosine kinase inhibitors only showed limited efficacy on residual GGNs after the surgery of major lesion(s) harboring *EGFR* mutation [26]. Although combination with a radiomics model that can predict *EGFR* mutation status could further improve treatment efficiency, the overall response rate was 53.8%, which happens to be consistent with the rate of *EGFR* mutation in East Asian populations [27]. The significant mutational heterogeneity across different lesions of MPLC in our study could account for the limited response rate to EGFR inhibitors. It is reasonable to infer that a significant reduction in diameter may only be observed in residual GGNs harboring sensitive EGFR mutations. A previous case report showed that the combination of osimertinib and alectinib resulted in disease stability in a patient diagnosed with MPLC harboring distinct *EGFR* and *RET* alterations [28]. However, it should be noted that the available evidence is insufficient to support the use of routine targeted therapy in MPLC.

In recent years, immunotherapy has reshaped the therapeutic landscape for patients with lung cancer. Nevertheless, whether this strategy can also be applied to MPLC remains unexplored. In our study, the TMB and PD-L1 expression were relatively low both in MPLC and SN, in accordance with the high prevalence of early-stage lung cancer in our cohort. Previous studies have shown that MPLC often exhibited low TMB, low PD-L1 expression, and heterogeneous immune cell infiltration [23, 29]. It is noteworthy that disparities in immunological features have also been documented between MPLC and SN. The PD-L1 expression and the density CD8^+^ tumor-infiltrating lymphocytes of MPLC were significantly lower than those of traditional lung cancer [12]. Additionally, more immune-related biological processes and pathways, such as B cell activation and the IL-17 signaling pathway, were identified in the MPLC cohort[14]. Furthermore, high mutation rate of *EGFR* in East Asian populations together with the inert tumor immune microenvironment in lesions presenting as pGGN collectively implied a heterogeneous and limited response to immunotherapy in patients with MPLC [30]. Several small-scale clinical studies have revealed that no obvious reduction in diameter was observed after receiving immunotherapy in most high-risk synchronous GGNs [31, 32]. Moreover, the heterogeneous drug response to neoadjuvant immunotherapy in different lesions of MPLC has been reported in a patient presenting with one solid nodule and two subsolid nodule [33]. Multi-omics analyses indicated the proportions of tissue-resident memory T cells and CD8^+^ T cells varied greatly between responding and non-responding lesions and impaired T lymphocyte immunity may lead to the inferior response to immunotherapy [33]. Altogether, perioperative immunotherapy may be not suitable for patients with MPLC due to the inert tumor immune microenvironment.

Given the crucial role of T cells in mediating the antitumor response, TCR sequencing could provide us with an opportunity to comprehensively assess the activation status of immune system. The higher TMB exhibits a trend associated with increased TCR clonality in both cohorts, supporting the critical role of somatic mutations in boosting tumor immunogenicity and eliciting T cell responses. The similar positive correlation was observed in American smokers, implying that the association between TMB and TCR clonality may not be influenced by ethnicity or tobacco exposure [18]. Besides the previously mentioned heterogeneity in the mutational landscape, considerable intrapatient and interpatient heterogeneity were also observed in TCR diversity and expansion [34]. Early carcinogenesis of lung cancer has been confirmed to be a gradual process shaped by host immune surveillance [35]. TCR repertoire displayed a gradual increase in diversity and a progressive decrease in expansion along with the progression of invasiveness from preneoplasia to invasive lung adenocarcinoma [36]. In this study, similar results were present in the MPLC cohort but not in the SN cohort. Furthermore, invasive lesions of MPLC harbored more diverse T cell infiltration than that of SN. The interaction between tumor and the host’s immune system is crucial for the control of tumor growth and spread. Therefore, as the tumor progresses from preneoplasia to IAC, the immune system is believed to gradually lose its ability to kill tumor cells and acquire suppressive or ineffective subsets of T cells [37]. Several studies have shown that TCR clonality was positively correlated with CD8^+^ T cells but negatively correlated with CD4^+^ T cells [36, 38]. Meanwhile, Dejima et al. found TCR clonality was not associated with T cell density in their study, suggesting that lower T cell clonality in the invasive lesions of MPLC may reflect reduced T cell expansion rather than T cell exclusion [36]. The most likely reason is that although the immune system recruited a greater number of T cells, the majority of them were bystander T cells and did not exert a suppressive effect on the tumor [39, 40]. Taken together, MPLC may represent a distinct subtype of lung cancer characterized by unique pathogenesis and immune response patterns, rather than simply being a combination of multiple solitary lung cancer nodules. Notably, differences in TCR repertoire between the two cohorts could be masked by the inclusion of MIA and lung adenocarcinoma precursor lesions. One possible reason is that lesions at those stages have a limited capacity to invade through the basement membrane and release the tumor antigens into the immune microenvironment [41].

Tumor-specific neoantigens derived from somatic mutations could induce T cell activation and provide a critical link between tumor and adaptive immune system [42]. Therefore, the marked heterogeneity of TCR repertoire in different lesions of MPLC could be the result of the interaction between diverse neoantigens in each lesion and shared immune contexture. The impact of shared environmental exposure and immune contexture on the occurrence and development of multiple lesions of the same patient may make them share a degree of similarity in biological features. Indeed, unlike the remarkable heterogeneity in the mutational landscape, it was recently shown that the DNA methylation patterns of multiple lesions of the same patient were very similar to each other [14]. In this study, lesions of the same patient always share a certain proportion of TCR clones. More importantly, significant clonal expansion could be observed in all 100% shared TCR clones, indicating that shared TCR clones might reflect the immune commonality among lesions of the same patient and play a critical role in the antitumor immune response [34].

The interaction between tumor cells and host immune surveillance is a dynamic process, and TCR on the surface of T cells specifically recognizes the neoantigens presented by major histocompatibility complex molecules is the first step [41]. Previous studies have shown that the immune system could exert evolutionary pressure through the process of immunoediting to eliminate highly immunogenic tumor clones, and ultimately leads to the outgrowth of the fittest clones [35, 43]. Notably, the vast majority of lesions in the MPLC cohort are at the early stages of lung cancer manifesting as pGGNs, which have been widely considered as indolent, asymptomatic, slow-growing tumors and can remain clinically silent for years [44]. Therefore, the tumor microenvironment at this time is still in the equilibrium phase, where the adaptive immune system cannot eliminate tumor cells, but keeps them in a state of immune-mediated tumor dormancy [30, 45]. Similar to the selective expansion of dominant tumor clones, T cell populations may also experience evolutionary selection pressure and eventually lead to the selective survival and clonal expansion of neoantigen-specific T cells to maintain immune equilibrium, following the Darwinian rule of natural selection [46]. One recent study has indicated that universally present neoantigens across multi-regions of the same tumor should be targeted to maximize the potential for uniform T cell responses due to the presence of neoantigen intratumor heterogeneity [47, 48]. In light of these findings, the identification of shared TCR clones that were significantly expanded across multiple lesions of the same patient may have important implications for the development of therapeutic approaches targeting all lesions simultaneously to solve the treatment dilemma of MPLC. Preclinical work has suggested that adoptive therapy with neoantigen-specific TCR-transduced T cells could significantly inhibit tumor growth in the mice xenograft model [49]. Tebentafusp, a T cell receptor-bispecific molecule has been confirmed to confer a long-term survival benefit and been approved for previously untreated patients with metastatic uveal melanoma [49]. Taken together, our findings are important for deepening the understanding of MPLC. We hope that this study could inspire an increasing number of researchers to focus on and actively engage in scientific research on MPLC to move the field forward.

To the best of our knowledge, this is the first systemic study to reveal the existence of immune commonality among different lesions of the same patient with MPLC via TCR sequencing. However, there are still some deficiencies in our research. First, the retrospective and single-center nature may inevitably introduce some selection bias in this study, and further validation of these results in prospective multi-center cohorts is warranted. Second, the scarcity and small sizes of lesions hindered more comprehensive multi-omics analyses. Moreover, tumor-specific neoantigens responsible for TCR clonal expansion and the correspondence between neoantigens and tumor-specific T cells should be further investigated. More in-depth studies, especially functional studies using humanized animal models are warranted to elucidate the detailed mechanisms.

## Conclusion

In conclusion, significant differences in the mutational landscape, activation status of oncogenic signaling pathways, and TCR repertoire between MPLC and SN collectively implied that MPLC might not be a simple combination of multiple solitary lung cancer. More importantly, the significant clonal expansion of shared TCR clonotypes demonstrated the existence of immune commonality among different lesions of the same patient and provided a new idea for resolving the treatment dilemma of MPLC.

### Supplementary Information

Below is the link to the electronic supplementary material.

Supplementary file1 (DOCX 8607 kb)

Supplementary file2 (XLSX 12 kb)

Supplementary file3 (XLSX 12 kb)

Supplementary file4 (XLSX 12 kb)

## Data Availability

The datasets generated and/or analyzed during the current study are available from the corresponding author on reasonable request. Qualified researchers can apply for access to these data by contacting Naixin Liang at pumchnelson@163.com, submitting a brief proposal, and signing a data usage agreement.

## Abbreviations

AAH: Atypical adenomatoid hyperplasia
AIS: Adenocarcinoma in situ
ANOVA: Analysis of variance
BRAF: B-Raf
EGFR: Epidermal growth factor receptor
ERBB2: Erb-b2 Receptor tyrosine kinase 2
IAC: Invasive adenocarcinoma
IQR: Interquartile range
KRAS: Kirsten rat sarcoma viral oncogene
MAPK: The mitogen-activated protein kinase
MAP2K1: Mitogen-activated protein kinase kinase 1
mGGN: Mixed ground-glass nodules
MIA: Minimally invasive adenocarcinoma
MOI: Morisita overlap index
MPLC: Multiple primary lung cancer
MTOR: Mechanistic target of rapamycin kinase
NGS: Next-generation sequencing
PD-L1: Programmed cell death-ligand 1
pGGN: Pure ground-glass nodules
PI3K: Phosphoinositide-3-kinase
RMB10: RNA binding motif protein 10
RTK: Receptor tyrosine kinase
SN: Solitary lung cancer nodule
TCR: T cell receptor
TMB: Tumor mutation burden
TP53: Tumor protein p53

## References

[1] Siegel RL, Miller KD, Fuchs HE, Jemal A (2021). Cancer stat. CA Cancer J Clin.

[2] Hattori A, Takamochi K, Oh S, Suzuki K (2020). Prognostic classification of multiple primary lung cancers based on a ground-glass opacity component. Ann Thorac Surg.

[3] Tian H, Bai G, Yang Z, Chen P, Xu J, Liu T, Fan T, Wang B, Xiao C, Li C (2023). Multiple primary lung cancer: Updates of clinical management and genomic features. Front Oncol.

[4] Mansuet-Lupo A, Barritault M, Alifano M, Janet-Vendroux A, Zarmaev M, Biton J, Velut Y, Le Hay C, Cremer I, Régnard JF (2019). Proposal for a combined histomolecular algorithm to distinguish multiple primary adenocarcinomas from intrapulmonary metastasis in patients with multiple lung tumors. J Thorac Oncol.

[5] Liu Y, Zhang J, Li L, Yin G, Zhang J, Zheng S, Cheung H, Wu N, Lu N, Mao X (2016). Genomic heterogeneity of multiple synchronous lung cancer. Nat Commun.

[6] Wu C, Zhao C, Yang Y, He Y, Hou L, Li X, Gao G, Shi J, Ren S, Chu H (2015). High discrepancy of driver mutations in patients with NSCLC and synchronous multiple lung ground-glass nodules. J Thorac Oncol.

[7] Hill W, Lim EL, Weeden CE, Lee C, Augustine M, Chen K, Kuan FC, Marongiu F, Evans EJ, Moore DA (2023). Lung adenocarcinoma promotion by air pollutants. Nature.

[8] Robins HS, Campregher PV, Srivastava SK, Wacher A, Turtle CJ, Kahsai O, Riddell SR, Warren EH, Carlson CS (2009). Comprehensive assessment of T-cell receptor beta-chain diversity in alphabeta T cells. Blood.

[9] Luo H, Zu R, Huang Z, Li Y, Liao Y, Luo W, Zhou P, Wang D, Chen S, Li W (2022). Characteristics and significance of peripheral blood T-cell receptor repertoire features in patients with indeterminate lung nodules. Signal Transduct Target Ther.

[10] Han J, Duan J, Bai H, Wang Y, Wan R, Wang X, Chen S, Tian Y, Wang D, Fei K (2020). TCR repertoire diversity of peripheral PD-1(+)CD8(+) T cells predicts clinical outcomes after immunotherapy in patients with non-small cell lung cancer. Cancer Immunol Res.

[11] Kozower BD, Larner JM, Detterbeck FC, Jones DR (2020). Special treatment issues in non-small cell lung cancer: diagnosis and management of lung cancer, 3rd ed: american college of chest physicians evidence-based clinical practice guidelines. Chest.

[12] Yang Z, Zhou B, Guo W, Peng Y, Tian H, Xu J, Wang S, Chen X, Hu B, Liu C (2024). Genomic characteristics and immune landscape of super multiple primary lung cancer. EBioMedicine.

[13] Nong J, Gong Y, Guan Y, Yi X, Yi Y, Chang L, Yang L, Lv J, Guo Z, Jia H (2018). Circulating tumor DNA analysis depicts subclonal architecture and genomic evolution of small cell lung cancer. Nat Commun.

[14] Yu F, Huang X, Zhou D, Zhao Z, Wu F, Qian B, Wang Q, Chen J, Liang Q, Jiang Y (2023). Genetic, DNA methylation, and immune profile discrepancies between early-stage single primary lung cancer and synchronous multiple primary lung cancer. Clin Epigenetics.

[15] Yang H, Wang Y, Jia Z, Wang Y, Yang X, Wu P, Song Y, Xu H, Gu D, Chen R (2021). Characteristics of T-Cell receptor repertoire and correlation with EGFR mutations in all stages of lung cancer. Front Oncol.

[16] Postow MA, Manuel M, Wong P, Yuan J, Dong Z, Liu C, Perez S, Tanneau I, Noel M, Courtier A (2015). Peripheral T cell receptor diversity is associated with clinical outcomes following ipilimumab treatment in metastatic melanoma. J Immunother Cancer.

[17] Li Y, Li X, Li H, Zhao Y, Liu Z, Sun K, Zhu X, Qi Q, An B, Shen D (2020). Genomic characterisation of pulmonary subsolid nodules: mutational landscape and radiological features. Eur Respir J.

[18] Reuben A, Zhang J, Chiou SH, Gittelman RM, Li J, Lee WC, Fujimoto J, Behrens C, Liu X, Wang F (2020). Comprehensive T cell repertoire characterization of non-small cell lung cancer. Nat Commun.

[19] Mansfield AS, Murphy SJ, Peikert T, Yi ES, Vasmatzis G, Wigle DA, Aubry MC (2016). Heterogeneity of programmed cell Death ligand 1 expression in multifocal lung cancer. Clin Cancer Res.

[20] Altorki N, Wang X, Kozono D, Watt C, Landrenau R, Wigle D, Port J, Jones DR, Conti M, Ashrafi AS (2023). Lobar or sublobar resection for peripheral stage ia non-small-cell lung cancer. N Engl J Med.

[21] Chang JY, Lin SH, Dong W, Liao Z, Gandhi SJ, Gay CM, Zhang J, Chun SG, Elamin YY, Fossella FV (2023). Stereotactic ablative radiotherapy with or without immunotherapy for early-stage or isolated lung parenchymal recurrent node-negative non-small-cell lung cancer: an open-label, randomised, phase 2 trial. Lancet.

[22] Ma P, Fu Y, Cai MC, Yan Y, Jing Y, Zhang S, Chen M, Wu J, Shen Y, Zhu L (2017). Simultaneous evolutionary expansion and constraint of genomic heterogeneity in multifocal lung cancer. Nat Commun.

[23] Hu C, Zhao L, Liu W, Fan S, Liu J, Liu Y, Liu X, Shu L, Liu X, Liu P (2021). Genomic profiles and their associations with TMB, PD-L1 expression, and immune cell infiltration landscapes in synchronous multiple primary lung cancers. J Immunother Cancer.

[24] Cheng H, Guo Z, Zhang X, Wang XJ, Li Z, Huo WW, Zhong HC, Li XJ, Wu XW, Li WH (2023). Lack of evolutionary convergence in multiple primary lung cancer suggests insufficient specificity of personalized therapy. J Genet Genomics.

[25] Tsuboi M, Herbst RS, John T, Kato T, Majem M, Grohé C, Wang J, Goldman JW, Lu S, Su WC (2023). Overall survival with osimertinib in resected EGFR-mutated NSCLC. N Engl J Med.

[26] Cheng B, Li C, Zhao Y, Li J, Xiong S, Liang H, Liu Z, Zeng W, Liang W, He J (2021). The impact of postoperative EGFR-TKIs treatment on residual GGO lesions after resection for lung cancer. Signal Transduct Target Ther.

[27] Shi Y, Au JS, Thongprasert S, Srinivasan S, Tsai CM, Khoa MT, Heeroma K, Itoh Y, Cornelio G, Yang PC (2014). A prospective, molecular epidemiology study of EGFR mutations in Asian patients with advanced non-small-cell lung cancer of adenocarcinoma histology (PIONEER). J Thorac Oncol.

[28] Aredo JV, Diehn M, Berry GJ, Wakelee HA (2021). Targeted treatment of multiple primary lung cancers harboring distinct EGFR or RET alterations: a case report. Clin Lung Cancer.

[29] Izumi M, Sawa K, Oyanagi J, Noura I, Fukui M, Ogawa K, Matsumoto Y, Tani Y, Suzumura T, Watanabe T (2021). Tumor microenvironment disparity in multiple primary lung cancers: Impact of non-intrinsic factors, histological subtypes, and genetic aberrations. Transl Oncol.

[30] Chen K, Bai J, Reuben A, Zhao H, Kang G, Zhang C, Qi Q, Xu Y, Hubert S, Chang L (2021). Multiomics analysis reveals distinct immunogenomic features of lung cancer with ground-glass opacity. Am J Respir Crit Care Med.

[31] Xu L, Shi M, Wang S, Li M, Yin W, Zhang J, Zhu J, Jiang F, Xia W, Qiu N (2022). Immunotherapy for bilateral multiple ground glass opacities: an exploratory study for synchronous multiple primary lung cancer. Front Immunol.

[32] Wu F, Li W, Zhao W, Zhou F, Xie H, Shi J, Yu G, Fan J, Jiang T, Zhou C (2020). Synchronous ground-glass nodules showed limited response to anti-PD-1/PD-L1 therapy in patients with advanced lung adenocarcinoma. Clin Transl Med.

[33] Zhang C, Yin K, Liu SY, Yan LX, Su J, Wu YL, Zhang XC, Zhong WZ, Yang XN (2021). Multiomics analysis reveals a distinct response mechanism in multiple primary lung adenocarcinoma after neoadjuvant immunotherapy. J Immunother Cancer.

[34] Reuben A, Gittelman R, Gao J, Zhang J, Yusko EC, Wu CJ, Emerson R, Zhang J, Tipton C, Li J (2017). TCR repertoire Intratumor heterogeneity in localized lung adenocarcinomas: an association with predicted neoantigen heterogeneity and postsurgical recurrence. Cancer Discov.

[35] Hu X, Fujimoto J, Ying L, Fukuoka J, Ashizawa K, Sun W, Reuben A, Chow CW, McGranahan N, Chen R (2019). Multi-region exome sequencing reveals genomic evolution from preneoplasia to lung adenocarcinoma. Nat Commun.

[36] Dejima H, Hu X, Chen R, Zhang J, Fujimoto J, Parra ER, Haymaker C, Hubert SM, Duose D, Solis LM (2021). Immune evolution from preneoplasia to invasive lung adenocarcinomas and underlying molecular features. Nat Commun.

[37] Sivakumar S, Lucas FAS, McDowell TL, Lang W, Xu L, Fujimoto J, Zhang J, Futreal PA, Fukuoka J, Yatabe Y (2017). Genomic landscape of atypical adenomatous hyperplasia reveals divergent modes to lung adenocarcinoma. Cancer Res.

[38] Rudqvist NP, Pilones KA, Lhuillier C, Wennerberg E, Sidhom JW, Emerson RO, Robins HS, Schneck J, Formenti SC, Demaria S (2018). Radiotherapy and CTLA-4 blockade shape the TCR repertoire of Tumor-infiltrating T cells. Cancer Immunol Res.

[39] Simoni Y, Becht E, Fehlings M, Loh CY, Koo SL, Teng KWW, Yeong JPS, Nahar R, Zhang T, Kared H (2018). Bystander CD8(+) T cells are abundant and phenotypically distinct in human tumour infiltrates. Nature.

[40] Meier SL, Satpathy AT, Wells DK (2022). Bystander T cells in cancer immunology and therapy. Nat Cancer.

[41] Zhang C, Zhang J, Xu FP, Wang YG, Xie Z, Su J, Dong S, Nie Q, Shao Y, Zhou Q (2019). Genomic landscape and immune microenvironment features of preinvasive and early invasive lung adenocarcinoma. J Thorac Oncol.

[42] DuPage M, Cheung AF, Mazumdar C, Winslow MM, Bronson R, Schmidt LM, Crowley D, Chen J, Jacks T (2011). Endogenous T cell responses to antigens expressed in lung adenocarcinomas delay malignant tumor progression. Cancer Cell.

[43] Matsushita H, Vesely MD, Koboldt DC, Rickert CG, Uppaluri R, Magrini VJ, Arthur CD, White JM, Chen YS, Shea LK (2012). Cancer exome analysis reveals a T-cell-dependent mechanism of cancer immunoediting. Nature.

[44] Sato Y, Fujimoto D, Morimoto T, Uehara K, Nagata K, Sakanoue I, Hamakawa H, Takahashi Y, Imai Y, Tomii K (2017). Natural history and clinical characteristics of multiple pulmonary nodules with ground glass opacity. Respirology.

[45] Schreiber RD, Old LJ, Smyth MJ (2011). Cancer immunoediting: integrating immunity's roles in cancer suppression and promotion. Science.

[46] Han J, Yu R, Duan J, Li J, Zhao W, Feng G, Bai H, Wang Y, Zhang X, Wan R (2021). Weighting tumor-specific TCR repertoires as a classifier to stratify the immunotherapy delivery in non-small cell lung cancers. Sci Adv.

[47] McGranahan N, Furness AJ, Rosenthal R, Ramskov S, Lyngaa R, Saini SK, Jamal-Hanjani M, Wilson GA, Birkbak NJ, Hiley CT (2016). Clonal neoantigens elicit T cell immunoreactivity and sensitivity to immune checkpoint blockade. Science.

[48] Yap TA, Gerlinger M, Futreal PA, Pusztai L, Swanton C (2012). Intratumor heterogeneity: seeing the wood for the trees. Sci Transl Med.

[49] Wang QJ, Yu Z, Griffith K, Hanada K, Restifo NP, Yang JC (2016). Identification of T-cell receptors targeting KRAS-Mutated human tumors. Cancer Immunol Res.

[50] Hassel JC, Piperno-Neumann S, Rutkowski P, Baurain JF, Schlaak M, Butler MO, Sullivan RJ, Dummer R, Kirkwood JM, Orloff M, Sacco JJ (2023). 3-Year overall survival with tebentafusp in metastatic uveal melanoma. New Eng J Med.

