# Supplementary material for: Clonal expansion of shared T cell receptors reveals the existence of immune commonality among different lesions of synchronous multiple primary lung cancer
Source: Cancer Immunol Immunother. 2024 Apr 26;73(6):111. doi: 10.1007/s00262-024-03703-8 (PMC11052747; doi:10.1007/s00262-024-03703-8)
Supplement: Supplementary file 1 — Supplementary file1 (DOCX 8607 kb) [file 262_2024_3703_MOESM1_ESM.docx]

**Supplementary Information for**

**Clonal expansion of shared T cell receptors reveals the existence of immune commonality among different lesions of** **synchronous multiple primary lung cancer**

**Cancer Immunology, Immunotherapy (submitted in2024) - Yadong Wang et al.**

**Supplementary Information include:**

5 supplementary figures with legends


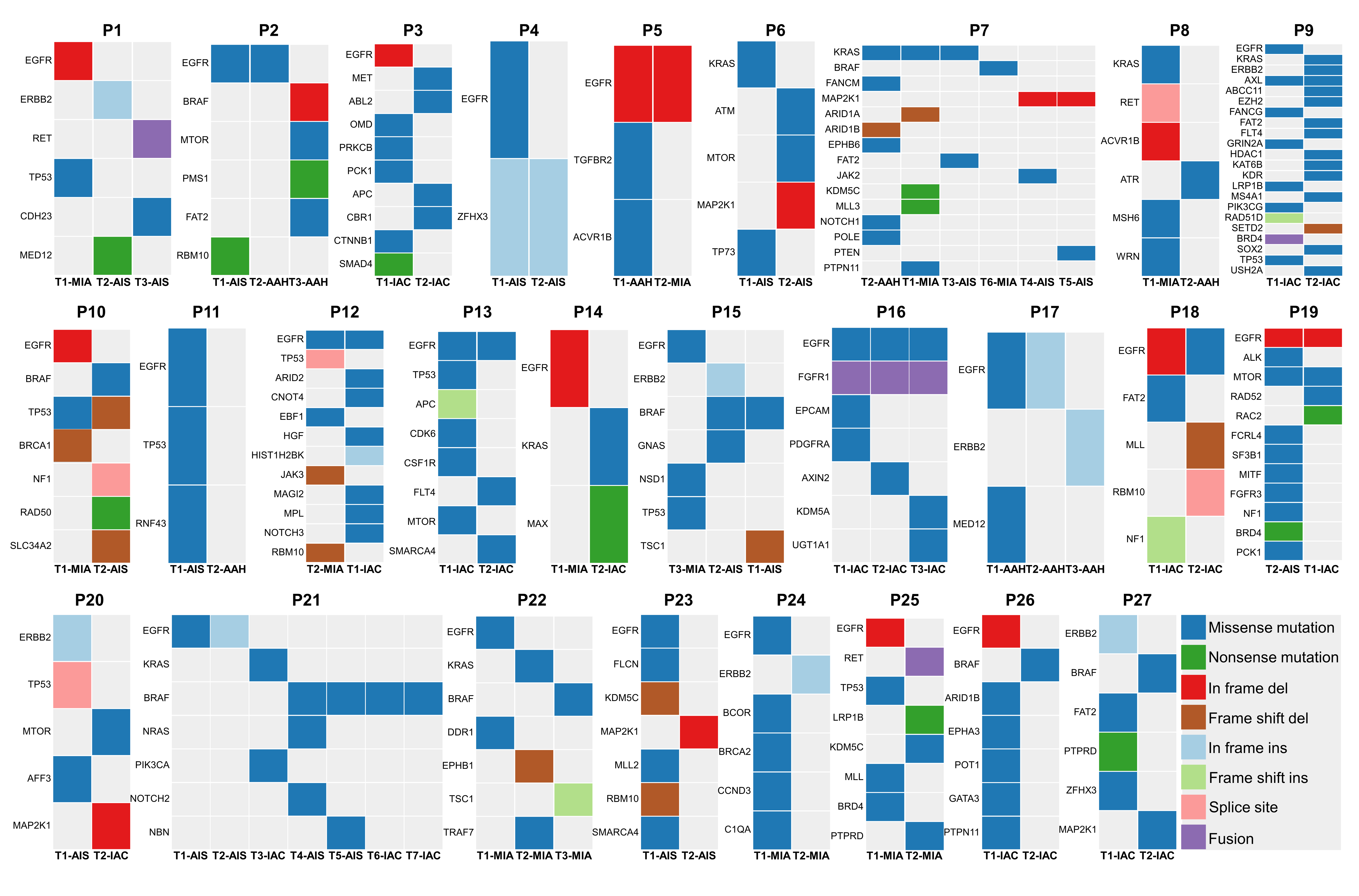


**Supplementary Figure. 1 The distribution of somatic mutations in lesions of the same patient in the MPLC cohort.** The distribution of non-synonymous somatic mutations of 69 lesions from 27 patients with MPLC is depicted in the heatmaps. Each column represents one lesion, and each row represents one gene. Grey represents wild-type gene, while mutated genes are shown in different colors according to the type of alterations. MPLC, multiple primary lung cancer.


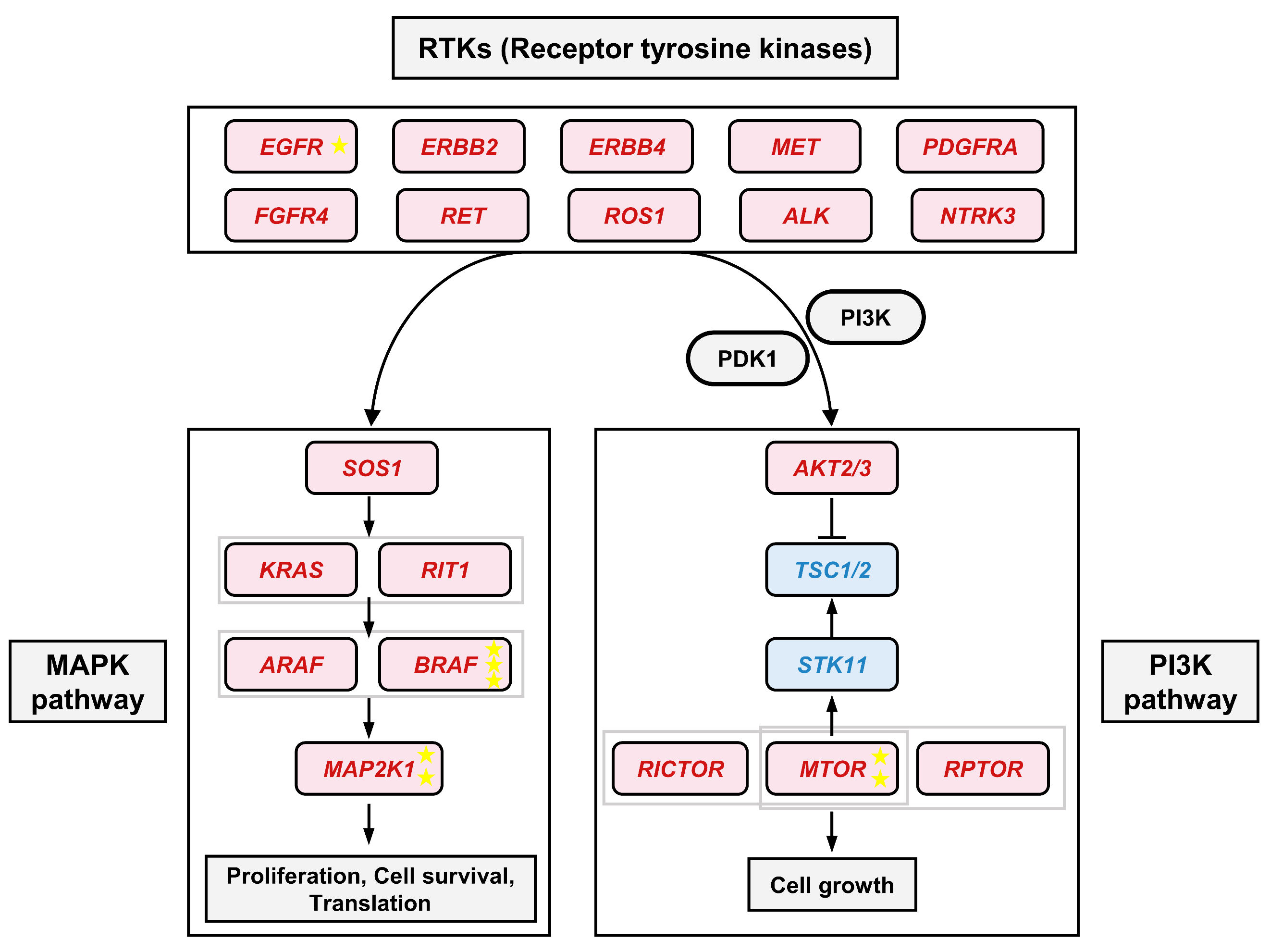


**Supplementary Figure. 2 Oncogenic signaling pathways containing genes with significantly different alteration rates between the two cohorts.** The BRAF, MAP2K1, and MTOR genes exhibit significantly elevated alteration rates in MPLC lesions and are involved in the MAPK and PI3K pathways, both of which lie downstream of RTK pathways. In contrast, lesions of SN displayed a significantly higher rate of EGFR mutation, which is an important component of the RTK pathways. The genes highlighted in red represent oncogenes, whereas those marked in blue denote tumor suppressor genes. The genes marked with yellow star(s) represent a significant difference in their alteration rates between the MPLC and SN cohorts. One star represents a p-value less than 0.05, two stars indicates a p-value less than 0.01, and three stars signifies a p-value less than 0.001. MPLC, multiple primary lung cancer; SN, solitary lung cancer nodule; MAPK, mitogen-activated protein kinase; PI3K, phosphoinositide-3-kinase; RTK, receptor tyrosine kinase. (Chi-squared/Fisher’s exact test)

**
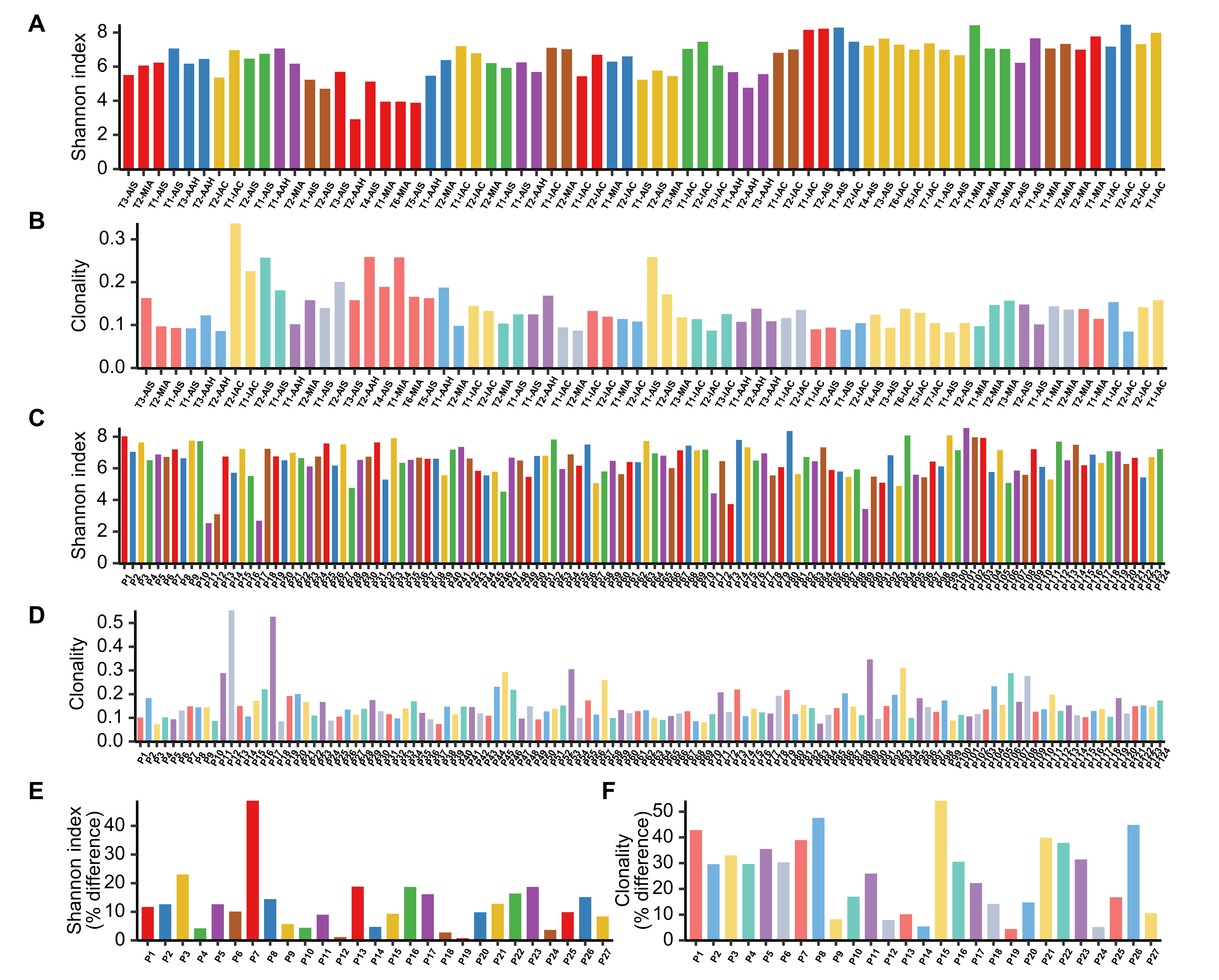
**

**Supplementary Figure. 3 The overall distribution of TCR repertoire and intrapatient differences of corresponding TCR repertoire metrics in the MPLC cohort.** The distribution of TCR Shannon index (A) and clonality (B) in the MPLC cohort. Each column represents one lesion, and adjacent columns labeled in the same color are derived from the same patient. The distribution of TCR Shannon index (C) and clonality (D) in the SN cohort. The intrapatient difference of Shannon index (E) and clonality (F) between different lesions of the same patient in the MPLC cohort. Each column represents one patient, and the vertical axis represents the relative magnitude of the intrapatient differences in TCR repertoire metrics. MPLC, multiple primary lung cancer; SN, solitary lung cancer nodule; TCR, T cell receptor.


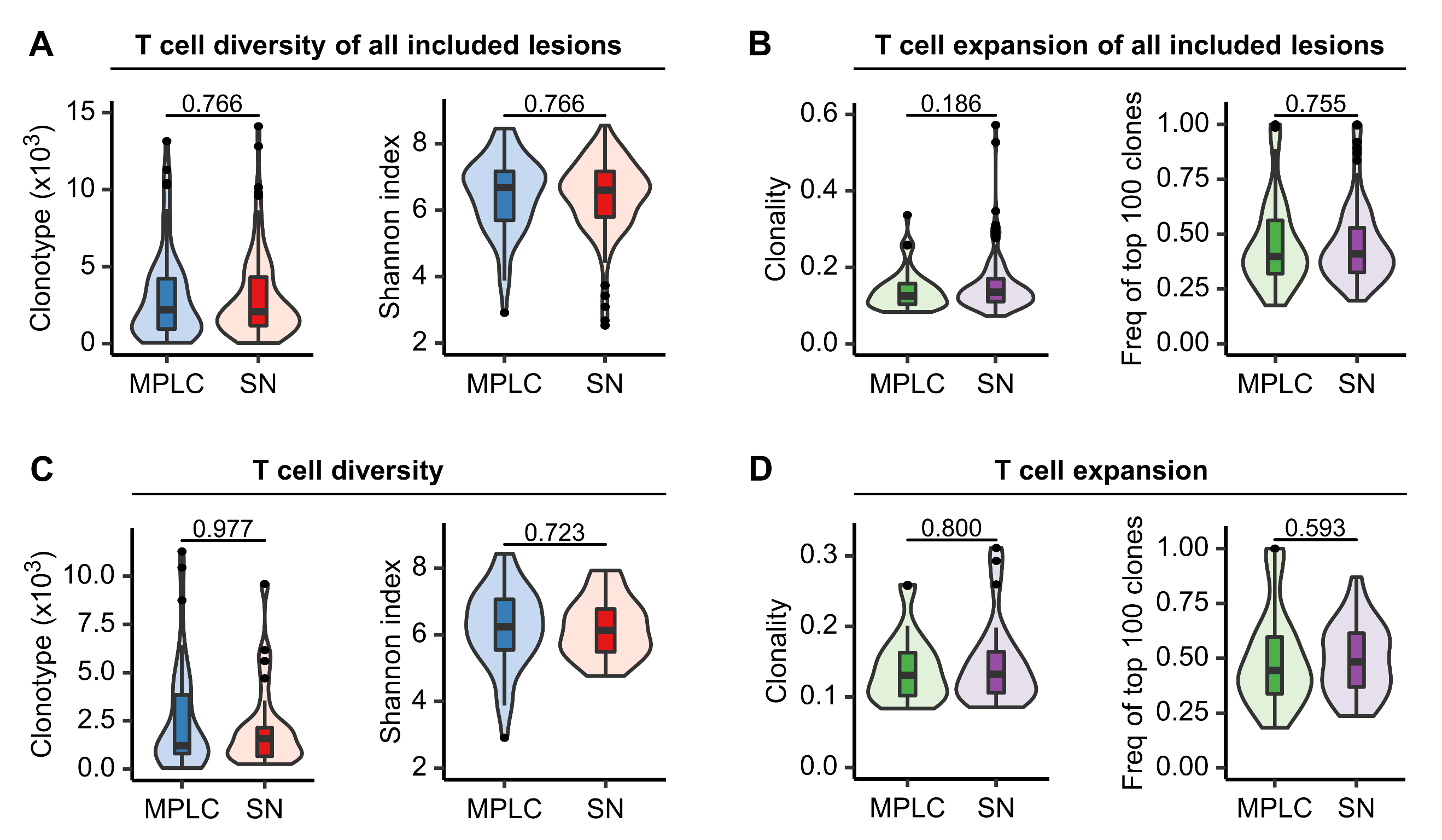


**Supplementary Figure. 4 Comparison of TCR repertoire** **between lesions of MPLC and SN.** The distribution and difference in TCR diversity (A) and expansion (B) between the MPLC and SN cohort when all lesions were included. The distribution and difference in TCR diversity (C) and expansion (D) between the MPLC and SN cohort when only lesions with AAH, AIS, and MIA were included. MPLC, multiple primary lung cancer; SN, solitary lung cancer nodule; TCR, T cell receptor; AAH, atypical adenomatoid hyperplasia; AIS, adenocarcinoma in situ; MIA, minimally invasive adenocarcinoma. (Mann-Whiney U test)


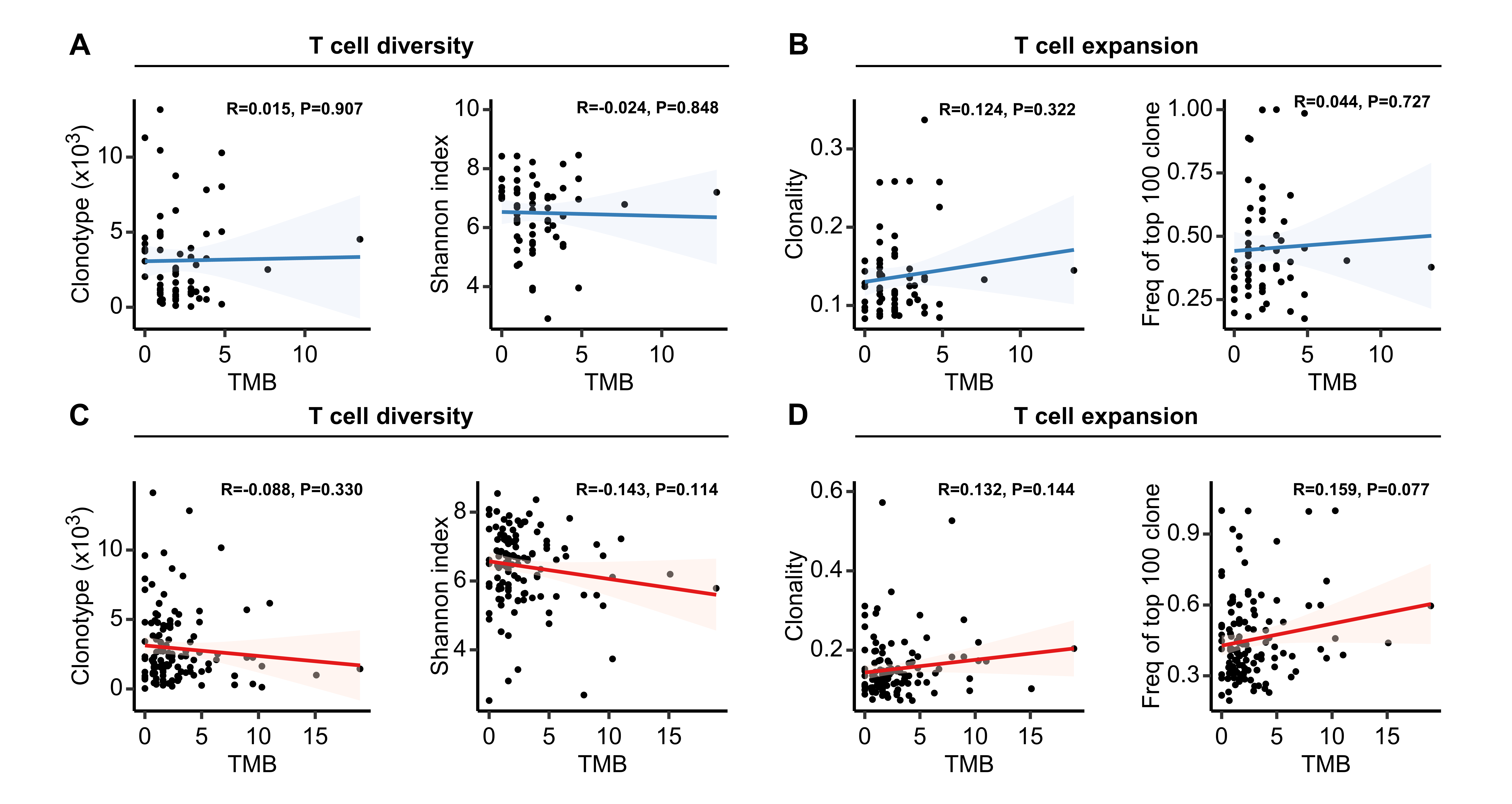


**Supplementary Figure. 5 The correlation between TCR repertoire and TMB in the MPLC and SN cohort.** Correlations between TCR diversity and TMB in the MPLC cohort (A). Correlations between TCR expansion and TMB in the MPLC cohort (B). Correlations between TCR diversity and TMB in the SN cohort (C). Correlations between TCR expansion and TMB in the SN cohort (D). The correlation coefficient (R) was assessed by Spearman’s rank correlation test. MPLC, multiple primary lung cancer; SN, solitary lung cancer nodule; TCR, T cell receptor; TMB, tumor mutation burden. (Spearman correlation analysis)
